# Towards a More Orderly Application of Proportionality to Patent Injunctions in the European Union

**DOI:** 10.1007/s40319-021-01139-6

**Published:** 2022-01-19

**Authors:** Rafał Sikorski

**Affiliations:** Professor, Faculty of Law and Administration, European Law Chair, Adam Mickiewicz University, Poznań, Poland

**Keywords:** Proportionality, Injunctive relief, Permanent injunctions, Abuse of rights, Equity, Patent assertion entities, Complex products

## Abstract

The intensity of IP protection has traditionally been determined by assessing the criteria that need to be satisfied for the protection to be granted, as well as the scope of rights and limitations of those rights. The enforcement stage and the remedies available to right holders have for long been, if not neglected, then certainly treated with lesser attention. The rise of aggressive litigation strategies, especially in the field of patents, has brought the enforcement stage to the forefront of the discussion about the proper level of IP protection in general and patent law in particular. Injunctions provide particularly strong leverage at the enforcement stage, allowing patentees in some cases to obtain royalties that exceed the value of the protected inventions. The principle of proportionality can play an important role as a check on excessive litigation strategies by patentees. Flexibility, however, comes at the price of uncertainty and unpredictability as to the outcome of patent disputes. Therefore, it is crucial to apply proportionality in an orderly and structured manner. For that purpose, the article identifies a set of factors that may be helpful in applying proportionality in patent disputes. That set of factors is well grounded in the traditions of the laws of the EU Member States.

## Background

International treaties concerning both copyright and industrial property protection have traditionally been preoccupied with ensuring a high level of protection for creations and inventions.^1^ In the European Union there has also been a steady tendency to achieve ever higher levels of intellectual property protection. For many years, at both the international and EU level, relatively little attention has been paid to enforcement of intellectual property rights. At the international level this changed with the adoption of the TRIPS Agreement,^2^ Part III of which deals with enforcement of intellectual property rights. A decade later, the then European Community adopted a directive that incorporated TRIPS Part III provisions and was solely dedicated to intellectual property enforcement.^3^ Interestingly, the enforcement measures were adopted with a view to ensuring that right holders had proper tools to combat piracy and counterfeiting, especially in the field of trademark and copyright law.^4^

TRIPS drafters could not have predicted or comprehensively responded to all the challenges facing right holders as a result of the rise of the internet and the shift from analog to digital.^5^ Neither could they have predicted the excessive litigation strategies that came with the ever more complex technologies protected by numerous rights, especially patents, and the growing role of entities preoccupied exclusively with asserting their patent portfolios in order to extort royalties significantly exceeding the value of the asserted patents.^6^ Since being adopted, a decade after TRIPS, Directive 2004/48 seems to have addressed at least some of the concerns resulting from the rise of the internet.^7^ Generally, however, the enforcement of various intellectual property rights poses different challenges for the right holders, infringers and public. Thus, whereas the enforcement of copyright on the internet has posed numerous challenges related to the protection of private life,^8^ the right to information^9^ or the freedom to conduct business,^10^ the aggressive and often excessive enforcement strategies related to patents are seen as a significant threat to innovation.^11^

The rise in excessive litigation in the field of patents has resulted from developments in the patent landscape, particularly in industries such as consumer electronics or computer and communication technologies.^12^ First, products in information and communication technology (ICT) and consumer electronics markets have become highly complex. They are made from multiple components, often supplied by various independent suppliers, and the technology involved is frequently protected by hundreds or even more patents. Second, multiple patents create “patent thickets”, making rights clearance costly and time consuming, if not altogether impossible.^13^ Third, large patent portfolios are acquired by entities that neither develop nor produce products and are exclusively preoccupied with asserting patents against manufacturers.^14^ Fourth, such entities often assert patents once the product has been designed and design costs have become sunk costs. The asserting entity can extract what is known as a “holdup value” for its patents, as the prospect of investments made in R&D activities being wasted enables the entity to demand higher – often excessively high – royalties.^15^ Finally, the right holder’s position vis-à-vis the infringer will only be further strengthened if the former is in a position to threaten the infringer with an injunction. In that case an injunction could later be used as leverage in licensing negotiations. Such an injunction would be even more painful for the infringer if it turned out to have resulted from infringement of a patent that reads only on a small and relatively unimportant component of a complex product.^16^

Although both TRIPS Part III provisions and Directive 2004/48 were designed with the purpose of providing the right holder with effective enforcement mechanisms, both also contain provisions that provide safeguards against excessive enforcement. The wording of Art. 3 of the Enforcement Directive is modelled after Art. 41 TRIPS. Thus, both acts require that measures be fair and equitable, that they avoid creating barriers to legitimate trade and that they provide safeguards against their abuse. Article 3(1) of Directive 2004/48 provides that enforcement measures be effective, dissuasive, and proportionate. Whereas the first two principles of the Enforcement Directive take the side of the patentee, proportionality implies the existence of a check on excessive use by the right holder of its rights, balancing the competing interests of right holders, infringers and possibly third parties or even the public; finally, it ensures some degree of flexibility and discretion at the enforcement stage.

This paper aims to analyze how the principle of proportionality affects or should affect the conditions under which injunctive relief in patent law is granted to patentees. Directive 2004/48 requires Member States to ensure that courts have the authority to grant injunctive relief. However, it does not require that they use that authority every time that they find an infringement.^17^ Interestingly, though the Enforcement Directive was adopted in 2004, the EU Member States’ courts do not seem to be very eager to embrace its foundational principles, especially the principle of proportionality. Whereas, generally, proportionality requires at least some degree of flexibility and discretion, most Member States still do not envisage room for such discretion.^18^ Consequently, courts in the majority of EU Member States, when faced with patent infringement disputes, are likely to grant injunctions (almost) automatically. Even in Member States that allow their courts some degree of discretion in granting injunctive relief, such flexibility is either never used or is used only very rarely.

The approaches towards proportionality vary both within the academic world and among the members of the judiciary. Some view proportionality as a mere formal tool that allows for the balancing of multiple and competing interests. Others claim that proportionality only enables the courts to promote their own policy choices rather than apply the law as it was written.^19^ This author subscribes to the view presented by R. Merges in *Justifying Intellectual Property* that proportionality is one of the highly important and useful organizing principles for the design and application of intellectual property law in general and patent law in particular.^20^ It is also crucial in the enforcement of patent law, especially for those industries where technologies are increasingly complex and patent thickets get ever denser. Most importantly, however – as will be shown below – proportionality does not need to be equated with unpredictability of results. Rather, it can be applied in an orderly manner, leading to both greater flexibility of injunctive relief and greater predictability of results.

This paper is divided into five sections. This background section is followed by Sect. 2 which will be devoted to the role played by proportionality in EU law. It will discuss the origins of the principle in constitutional or administrative law and the role that proportionality plays or could play in the field of private law. It will also discuss whether proportionality could be recognized more generally as a principle governing the scope, intensity, and enforcement of intellectual property protection. Section 3 will discuss proportionality as a principle of IP law. It will also discuss how proportionality is introduced at international, EU and national levels. Section 4 will look at the case law of the Court of Justice of the European Union (CJEU) that deals with the application of proportionality in the realm of copyright. This case law is particularly interesting because the CJEU considered proportionality when balancing fundamental rights to decide whether injunctions against intermediaries could be granted. The CJEU also looked through the lens of proportionality at measures other than the remedies proper as well as at the remaining remedies such as damages.^21^ Section 5 will deal with the application of the proportionality principle to injunctive relief in patent law. This will be presented against the background of various developments in technology markets such as excessive enforcement practices by patent assertion entities (PAEs), patent thickets, patent holdup, parallel innovation, and the complex nature of products. Finally, an attempt will be made to list factors that should be considered when assessing proportionality of injunctive relief in patent law. Having a list of factors allows for a more orderly and thus more predictable application of proportionality. Section 5 will be followed by concluding remarks.

## Proportionality and EU Law

Before analyzing the application of the proportionality principle to patent enforcement, we will take a closer look at the origins of and the role that proportionality plays in EU law. Proportionality – alongside supremacy, direct effect and liability for breach of EU law – stands out as one of the most significant principles of EU law.^22^ Proportionality was initially introduced to the EU legal order from the common constitutional traditions of the laws of the Member States by the CJEU.^23^

The underlying idea behind proportionality is that the exercise of power (or rights) should be subject to some internal limitations capable of safeguarding against excessive behavior on the part of the party holding power (right holder).^24^ When applied in the realm of constitutional law, it has the effect of limiting the ability of the state to restrict individual or local government rights.^25^ Similarly, in the sphere of administrative law, proportionality operates as a check against the abuse of power by administrative authorities.^26^ There is also room for proportionality in private law, including IP. There, proportionality may very well justify the doctrine of abuse of rights and limit how rights are exercised.^27^

In European Community (EC) law, proportionality emerged in the 1970s when Member States were attacking EC legislation for failing to comply with fundamental rights enshrined in national constitutions. In *Internationale Handelsgesellschaft,* Germany claimed that it could review EC legislation for compliance with national provisions on fundamental rights.^28^ The Court of Justice was of a different opinion. It insisted that it had sole competence to decide upon the legality of EC legislation. To protect the supremacy and direct effect of EC law the Court of Justice ruled that the EC legal order also protected fundamental rights and allowed those rights to be limited only when they would not be disproportionately affected by EC legislation. The Court of Justice stated that both the protection of fundamental rights and proportionality were derived from common constitutional traditions of the Member States and constituted general principles of EC law.

Nowadays, the CJEU also applies proportionality when it assesses measures by Member States that constitute restrictions on fundamental economic freedoms guaranteed under the Treaty on the Functioning of the European Union (TFEU).^29^ The CJEU, when assessing the compatibility of such measures with EU law, would first look at whether the purpose of such restrictions was lawful in view of the provisions of the TFEU. Then, the Court would proceed to assess the proportionality of the measure and analyze whether such a measure was capable of achieving that lawful purpose and whether that lawful purpose could be achieved in a less restrictive manner. The TFEU allows Member States to restrict fundamental freedoms where necessary for the protection of intellectual property. In the past, the CJEU often had to deal with provisions of national IP law and their compliance with provisions regulating fundamental freedoms.^30^

The role of proportionality as one of the most crucial principles of EU law is now recognized in the provisions of EU primary law in Art. 5 of the Treaty on European Union (TEU) and Protocol 2 thereof. Proportionality serves a correcting function in respect of the exercise of EU jurisdiction as well as in Art. 52 of the Charter on Fundamental Rights, which provides that limitations of fundamental rights by Member States and the EU are to be assessed under the proportionality principle. This express recognition of the role of proportionality is not surprising. The EU legal order has evolved greatly over time and its foundational documents are now regarded as resembling a constitutional order. With the embrace of fundamental rights there is often a need to balance the competing and conflicting rights. This is where proportionality comes into play.

How much room there is for proportionality analysis depends on the degree of harmonization at EU level. When a common regulatory regime is in place or when a given area of law has been subjected to harmonization there will be less room for balancing and thus for the application of proportionality. This can be seen in copyright, which – at least with respect to the scope of exclusive rights and their limitations and exceptions – is subject to a significant degree of harmonization. The EU directives on copyright already reflect the delicate balancing of the interests of users and copyright holders. Thus, the need for a different balance between competing interests is limited.^31^ The framework of the copyright directive is already based on balancing competing fundamental rights – especially IP rights, and the right to privacy, information, free speech and freedom to conduct business.^32^

Analysis of the EU proportionality principle allows one to make some more general comments as to how that principle functions. First, it seems that the scope for application of the proportionality principle in EU law is quite broad. It is used to assess the measures of the EU institutions and the Member States, and can also be used when interpreting EU law or national regulations implementing EU law in disputes between private parties. Second, the proportionality standard as applied in particular circumstances might vary. For example, Souter suggests that, when adjudicating on measures taken by the EU, a manifestly disproportionate standard is applied, whereas when the acts of Member States are under scrutiny, the CJEU refers to a stricter proportionality analysis.^33^ Third, proportionality analysis requires first that the suitability or appropriateness of a given measure for a particular lawful purpose is analysed. Then the measure is assessed to see if it is the least restrictive one for achieving a particular lawful purpose. Proportionality also implies a cost and benefit analysis. Fourth, the proportionality analysis may also require the proper balancing of fundamental rights or – in some cases – fundamental rights and fundamental economic freedoms. Fifth, proportionality always comes with a degree of flexibility and discretion, which are present at all levels as the principle is applied. Finally, proportionality was certainly used very instrumentally by the CJEU. Its recognition as a principle of EU law (together with the protection of fundamental rights) allowed the CJEU to prevent Member States’ courts from reviewing EU legislation. And it was also used as a tool to further economic integration.

## Proportionality and IP Law

### Proportionality as a General Principle for IP Law

In the IP context, proportionality has been analyzed primarily with regard to remedies. It has come to be embraced, initially by IP scholars, but now increasingly by the courts as well, as a tool to ensure much required flexibility, especially in the context of patent enforcement. However, the role of proportionality does not have to be limited to remedies and enforcement: proportionality is a broader concept that can also be relevant for other areas of intellectual property.

In his book *Justifying Intellectual Property*, Merges sees proportionality as one of the basic concepts of intellectual property law, alongside efficiency, public domain and dignity.^34^ All of these, referred to as mid-level principles, are concepts that run through a particular field of law and involve more detailed rules, doctrines or institutions.^35^ Merges sees proportionality as a concept that influences the way we look at criteria for obtaining protection, the scope of the right and, more generally, the design of IP rights, that is the scope of exclusivity together with exceptions and limitations. For Merges, the scope of an IP right should reflect the degree of creativity or innovativeness of the author or inventor.

Merges sees proportionality as an organizing principle with respect to IP law more generally. The basic idea of proportionality in IP law is that the right should not grant more power than the holder actually deserves. In particular, Merges sees a danger of disproportionate reward to the holder when the latter exercises control over larger markets than those that would be justified by the scope of its right.^36^ He also provides ample examples of how proportionality works within IP law generally and patent law in particular. When discussing non-obviousness, for example, Merges notes that a patent may only be granted when the inventor’s contribution is of some significance compared with the prior art. Since the patentee is about to receive an exclusive property right to the invention, its technical contribution must be proportionately significant.^37^ Proportionality also dictates what should be disclosed by the patentee in relation to its claims. Merges notes that a patent cannot claim more than it teaches.^38^

Merges also shows how proportionality could contain excessive litigation practices and refers to the situation of the manufacturer of a complex product, who has already invested heavily in bringing its product to the market, and a patentee who happens to hold a patent reading on one of many components of that complex product.^39^ He observes that, if patent law allowed injunctions to be granted automatically whenever a patent was infringed, the patentee would obtain enormous leverage in its negotiations with the manufacturer of the complex product, such as would allow the former to claim royalties far in excess of the value of its patent.^40^

### Proportionality and the Purpose of IP Protection

In the European Union, the purpose of intellectual property protection is revealed in the relevant directives and regulations. This is true for copyright, designs, and trademarks, as well as for scattered patent law related legislation. The protection of intellectual property rights, particularly in the fields of patent and copyright law, is aimed at stimulating innovation and creativity. It is also often assumed in EU legislation that ensuring a high level of IP protection is a means of attracting more R&D spending and consequently more innovation.^41^ However, designing IP laws requires numerous other interests to be taken into account, such as protection of health, access to cultural heritage, access to education and information, and free speech. Many of these interests are protected as human rights.^42^ These various policy concerns are also addressed by the EU legislator.

Drafting IP laws in a way that would stimulate more R&D spending and consequently more innovation and progress is a complex task. Just raising the levels of protection, by creating new rights or strengthening exclusivity may not yield the desired results.^43^ Sometimes creating an innovation-friendly ecosystem will require implementing limitations and exceptions, such as the research exception in patent law, which ensures access to knowledge. On other occasions it will require expressly removing certain intangibles from the realm of protection.^44^ In the case of remedies, considerations of maintaining incentives in the IP system to stimulate innovation and creativity are also important. The prospect of excessive remedies can deter innovators and chill innovation. This is true for both damages and injunctive relief when that relief may create leverage that allows excessive royalty payments to be extorted.

Too strong a system of IP protection may not be optimal from the perspective of stimulating innovation.^45^ This applies equally to the remedies available to holders of IP rights. If remedies are granted in a manner that allows exorbitant royalty payments to patent holders, they are not suitable for achieving the goals of promoting innovation as set out by the EU legislator. This is exactly where the principle of proportionality should intervene.

### Injunctions and Proportionality in International, EU and National Law

#### Proportionality and International Instruments

At international level, TRIPS explicitly recognizes a patentee’s right to demand an injunction from judicial authorities against an infringer.^46^ Interestingly, though it was concluded before the age of excessive patent litigation, it also recognizes the need for a degree of flexibility to be available to judicial authorities in administering injunctive relief.

TRIPS Part III provides that remedies should be applied “in such a manner as to avoid the creation of barriers to legitimate trade and to provide for safeguards against their abuse” (Art. 41.1 TRIPS). It also requires the remedies to be “fair and equitable” (Art. 41.2 TRIPS). References to “safeguards against abuse” and “fairness and equity” in international law are treated as references to the principle of proportionality, good faith, due process, and non-discrimination.^47^

TRIPS also explicitly recognizes specific cases when injunctions could be denied. *Firstly*, Art. 44(2) contains a general provision that allows an injunction to be denied (but only on condition that declaratory judgments and adequate compensation be available) when issuing one would be inconsistent with a member’s law. This would be the case, for example, when seeking an injunction could constitute an abuse of right under a member’s national law. *Secondly*, Art. 44(1) provides that TRIPS members are not required to grant an injunction against those who order or acquire protected subject matter prior to knowing or having reasonable grounds to know that dealing with such subject matter would entail infringement. An injunction could thus be denied if patentees choose to assert patents against manufacturers of complex products rather than against manufacturers of components. *Thirdly*, Art. 40 also recognizes that exercising intellectual property rights may have an adverse impact on competition as a result of certain licensing practices or unilateral actions by patentees who seek injunctions.

Post-TRIPS, a number of other international trade agreements have introduced the principle of proportionality to chapters dealing with the enforcement of IP rights. For example, the Comprehensive and Progressive Agreement for Trans-Pacific Partnership (CPTPP), which incorporated the provisions of the Trans-Pacific Partnership Agreement (TPP), expressly requires (in Art. 18.71(5)) account to be taken of the need for proportionality between the seriousness of the infringement and the interests of third parties.^48^ In the same spirit, the EU-Canada Comprehensive Economic and Trade Agreement (CETA) provides that procedures for the enforcement of intellectual property rights should inter alia be *fair and equitable* and “shall be applied in such a manner as to avoid the creation of barriers to legitimate trade and to provide for safeguards against their abuse”.^49^ It also provides that parties “shall take into account the need for proportionality between the seriousness of the infringement, the interests of third parties, and the applicable measures, remedies and penalties”.^50^

#### Proportionality and EU Member State Laws

Though the introduction of proportionality in Art. 3 of Directive 2004/48 could seem novel, the idea that rights should not be exercised in an excessive and abusive manner is not new to the legal systems of the EU Member States. The abuse of rights doctrine started to develop in the 19th century, and now most EU Member States recognize it in one form or another.^51^ It is a broad concept, which operates as a mechanism for correcting the abusive exercise of rights by enabling control of their exercise through concepts such as good faith, fair dealing, morality, proportionality, reasonableness and the socioeconomic functions of rights.^52^

Though particular formulations of the doctrine may differ among Member States, the abuse doctrine generally covers four different instances of abusive exercise of rights.^53^ Firstly, rights might be abused when exercised with the sole purpose and intention of harming the other party. Secondly, a right is abused when its exercise results in disproportionate harm to the other party in comparison to the benefits that accrue from such exercise to the right holder. Thirdly, it is considered abusive to exercise a right in a manner that contradicts the reasonable expectations of other parties created by the right holder. Fourthly, rights might also be abused when they are exercised contrary to their function or socioeconomic purpose.

These four types of abuse are broad and flexible, thus capable of catching numerous instances where various private rights, including intellectual property rights, are exercised wrongfully or in a way that is unworthy of protection. The above instances of abuse seem to be capable of addressing some of the concerns resulting from excessive patent litigation. For example, an injunction, if sought by a PAE, might cause significant harm to the infringer, while the benefits to the PAEs are unlikely to be of any additional significance. In a recent paper, A. Ohly showed – with respect to unfair competition and trademark laws in Germany – how abusive enforcement could be curtailed by Art. 242 of the German Civil Code (BGB), which safeguards against abuse of rights.^54^ However, in practice, especially patent law practice, the abuse of rights doctrine has been applied very cautiously. It is generally viewed as an exceptional mechanism for correcting the exercise of rights. Certainly, its open-ended character is its greatest advantage, while at the same time posing the most risks. However, as will be shown below in Sect. 5, these risks can be addressed by providing a set of factors that would help the proportionality analysis to proceed in a more orderly and thus predictable manner.

## Proportionality and Remedies in EU Copyright Law

The need to balance fundamental rights when deciding upon the availability of certain relief measures, including injunctions, to intellectual property holders has already been addressed by the CJEU in a series of copyright cases. The CJEU had to decide about the availability of certain remedies against internet service providers (ISPs). There the Court made very explicit references to the principle of proportionality, stating that regard had to be had to the principle when balancing various conflicting fundamental rights.

The scope of discretion available to the Court has been somewhat limited because of harmonization in the areas of E-commerce, data protection and copyright law. The regulatory framework in the harmonized areas already reveals some of the preferences of the EU legislator for a particular outcome of the fundamental rights balancing exercise. The scope of injunctive relief against an intermediary, which – by virtue of Art. 8(3) of Directive 2001/29/EC^55^ and Art. 11 of Directive 2004/48 – should be provided to the right holders, is restricted by the requirements imposed by Art. 15 Directive 2000/31,^56^ which requires that ISPs may not be subject to general monitoring obligations. The case law on measures available against intermediaries is illustrative of how the Court proceeds when balancing various fundamental rights and how proportionality affects that balancing exercise.

In *Promusicae* the reference for a preliminary ruling came in proceedings between Promusicae, a non-profit organization of producers and publishers of musical and audiovisual recordings, and Telefonica, an internet access provider.^57^ Promusicae petitioned the Madrid court to order Telefonica to release the identity and physical addresses of private users who had shared phonograms via the KaZaA file exchange program in violation of the exploitation rights held by Promusicae members.^58^ The referring court asked the CJEU whether in light of EU law – particularly Directives 2000/31, 2001/29 and 2004/48, and Arts. 17(2) and 47 of the EU Charter of Fundamental Rights (hereinafter, the Charter), the internet access provider was obliged to release the identity and personal addresses of its customers when such information was sought in civil proceedings.

The CJEU observed that the case involved the need to balance conflicting fundamental rights, namely: the right to property, the right to effective judicial remedy, and the right to respect for private life and personal data.^59^ The Court stated that a fair balance between conflicting rights had to be struck having regard to the general principle of EU law, namely the principle of proportionality.^60^ Here the CJEU concluded that mechanisms allowing different fundamental rights to be balanced were contained in the provisions of the directives referred to above as well as in Directive 2002/58.^61^ The provisions of those directives – according to the CJEU – did not require disclosure of the identity and personal addresses of internet users in civil proceedings.^62^

In *Scarlet Extended*, the reference for a preliminary ruling came in proceedings between SABAM, a rights management company representing authors, composers and editors of musical works, and Scarlet, an ISP that provides customers with access to the internet while not offering other services such as downloading or file sharing.^63^ SABAM claimed that Scarlet’s customers were using Scarlet’s services to download files over peer-to-peer networks, which – in SABAM’s opinion – constituted copyright infringement.^64^ In the Brussels first instance court, SABAM obtained an injunction against Scarlet. The court ordered Scarlet to bring its infringement to an end by preventing its users from sending and receiving musical works from SABAM’s repertoire.^65^

Scarlet challenged the decision, claiming that the injunction imposed a *de facto* general monitoring obligation in respect of all electronic communications between users of the ISP’s services, contrary to Art. 15 of Directive 2001/31.^66^ Scarlet also contended that the injunction was granted in breach of provisions on the protection of personal data and secrecy of communications.^67^ The Brussels Court of Appeal referred the matter to the CJEU, asking whether such a general injunction, which required the ISP to monitor all incoming and outgoing communications of all of its customers for an unlimited period, exclusively at its expense, was consistent with Directives 2000/31, 2001/29, 2004/48 and 2002/58.^68^

The CJEU found the obligation to carry out preventive monitoring of all communications by all customers of the ISP to be inconsistent with the prohibition on general monitoring in Art. 15 of Directive 2000/31.^69^ It also stated that such a general monitoring obligation in respect of all communications by all users would be incompatible with Art. 3 of Directive 2004/48 as disproportionate, unfair and excessively costly.^70^ Interestingly, though the CJEU could have stopped at this point, it went on, balancing the fundamental rights relevant in this case, namely the right to intellectual property, the freedom to conduct business, the right to protection of personal data, and the freedom to receive and impart information, seemingly with the intention of providing additional justification at the level of fundamental rights. The CJEU began its fundamental rights analysis by recognizing that the injunction was concerned with the protection of copyright, that is property within the meaning of Art. 17(2) of the Charter, adding, however, that copyright was not inviolable and that its protection was not absolute.^71^ The CJEU considered the monitoring obligation imposed on the ISP complicated and costly to the extent that it was regarded as a serious infringement of the freedom to conduct business.^72^ Finally, the CJEU also took the fundamental rights of third parties into account and found that the monitoring obligation would result in serious violations of the users’ freedom to receive and impart information, and the protection of personal data.^73^

In *UPC Telekabel* the request for a preliminary ruling was made in proceedings between UPC Telekabel, an ISP providing internet access to its customers, and Constantin Film and Wega, two film production companies, concerning an application for an interim injunction against UPC Telekabel. The film production companies demanded that UPC Telekabel be ordered to block its customers’ access to a website that made some of the films of Constantin Film and Wega available to the public without the consent of those companies.^74^ The order was issued by the Vienna Commercial Court and was later partly reversed by the Court of Appeal. The latter decided that UPC Telekabel could be ordered to block access, but had to remain free with respect to the means used.^75^ Consequently, UPC Telekabel could escape incurring coercive penalties if it could show that it had taken all reasonable measures to block such access.

After the case was further appealed to the Supreme Court, the CJEU was asked whether the injunction granted was compatible with the right to conduct business.^76^ The Court repeated its earlier case law and admitted that the decision required balancing the right to intellectual property, the freedom to conduct business, and the freedom of information of internet users in light of the principle of proportionality.^77^ The CJEU admitted that an injunction was indeed a limitation on the freedom to conduct business. It also explained that the right entailed the freedom to use all the economic, financial and technical resources within the control of an undertaking.^78^ It recognized that an injunction could have an impact on the organization of an intermediary and that it could require difficult and complex technical solutions to be taken; finally, it could also be costly.^79^

The CJEU recognized that although the measures limited the freedom to conduct business, they would not be capable of depriving UPC Telekabel of the very essence of that freedom.^80^ The CJEU observed that liability could be avoided if and when the intermediary was capable of showing that it had taken all necessary steps, particularly all reasonable steps, to block access.^81^ The Court added, however, that, when implementing the injunction, the ISP was obliged to ensure compliance with the freedom of internet users to information as guaranteed by Art. 11 of the Charter. Thus, the measures had to be targeted so that the users’ right to information would not be negatively affected by access to lawful content being blocked.^82^

In *McFadden,* the request for a preliminary ruling came in proceedings between a Mr McFadden, who provided anonymous access to a wireless local area network free of charge in the vicinity of his business, and Sony, a phonogram producer, whose recording was made available by a third party via the wireless network.^83^ Among the many issues raised, the question as to what measures McFadden was supposed to take to prevent recurrence of the infringement was particularly interesting. The CJEU was asked to consider the scope of an injunction that could be entered against the service provider in order to protect Sony’s rights to the phonogram, admitting that an injunction was likely to have an effect on the economic activity of the service provider and might also affect the freedom of the service’s users.^84^

The Court noted that McFadden could take three different measures: monitor all communications, terminate communications or password-protect communications.^85^ The first possibility was excluded outright as contrary to Art. 15 of Directive 31/2000.^86^ The second solution was considered to excessively restrict freedom to conduct business, especially if it involved a relatively limited copyright infringement.^87^ The third solution required only minor technical adjustments and could be considered as not damaging the essence of the freedom to conduct business.^88^ The Court also took third party rights into account and stated that the measure, as a strictly targeted one, did not restrict the service users’ freedom to information.^89^

In all the cases discussed above, the CJEU had to balance fundamental rights in light of the principle of proportionality. The Court leaves no doubt as to the obligation of the national courts to apply proportionality when interpreting and applying national laws implementing EU directives and when balancing various fundamental rights. In *Promusicae* and *Scarlet Extended*, it was relatively easy to balance the various rights. In fact, it had already been reflected in the regulatory framework of the various EU directives. However, in *McFadden* and especially *UPC Telekabel*, the balancing task was more demanding, and the decision required a more profound justification of the burden inflicted upon the ISP on the one hand and its customers on the other.^90^

It is worth noting that the CJEU does not perceive property as an inviolable right. It expressly accepts that property is not absolute and that it might be subject to limitations resulting from the existence of other competing rights. The decisions of the CJEU are also interesting as they shed some light on the way the CJEU approaches the freedom to conduct business and how that right is balanced with other fundamental rights. The CJEU admits that such freedom is affected negatively when the remedies result in additional costs or organizational burdens for the service provider. The question is one of degree. In *Scarlet Extended* the general monitoring obligation imposed on the ISP was perceived as an excessively onerous limitation of the freedom to conduct business. In *UPC Telekabel* however, the ensuing costs for the service provider were acceptable and justified in order to ensure adequate protection for copyright. Similarly, in *McFadden*, password-protecting – though requiring adjustments and consequently some additional costs – was acceptable.

The concerns addressed by the CJEU in *Promusicae*, *Scarlet Extended*, *UPC Telekabel* and *McFadden* sound quite familiar in the context of patent enforcement. An injunction certainly is a suitable remedy for protecting a patentee’s intellectual property. However, an injunction can also critically affect the infringer’s situation, leading to substantial costs associated with product redesign and removal of the product from distribution channels, in turn raising concerns that it could also significantly affect the infringer’s freedom to conduct business. These concerns require careful balancing in light of the principle of proportionality. The outcome of that balancing exercise – which is always fact-intensive – must take into account numerous other factors such as, inter alia, the nature of the protected invention, the behavior of both patentee and infringer, the commercialization strategy adopted by the patent holder, and the interests of both third parties and the public. Each of these specific factors requires careful assessment.

## Towards a More Orderly Application of Proportionality for Patent Injunctions

Patent law, just like all other fields of IP law, must adapt in order to respond to new challenges resulting from technological progress and rapidly evolving markets. These challenges might require some reassessment of protectability criteria, the scope of rights, and exceptions or amendments to enforcement mechanisms. Legislative changes usually lag behind the changing reality; therefore, building flexibility into the system is often a much better solution. In the case of enforcement, rigidity and an inability to tailor relief to the requirements of a particular case may harm some market participants, while providing others with undeserved and disproportionate gains.

However, flexibility always comes at a cost. Rigid or simplistic rules are at least predictable and usually produce results expeditiously. Flexible rules, on the other hand, are less certain, subject to discretion and generally slower in producing outcomes. These dangers are known to all participants in the debate on the role for proportionality in injunctive relief in patent law. This section intends to show that adopting a set of guiding factors that are consistent with the understanding of proportionality in EU law generally and in the broader sphere of IP law could assist the courts in the predictable application of proportionality while still having the benefit of flexibility. Proportionality is sometimes equated with depriving patentees of injunctive relief. Some suggest that it is the end of patents as exclusive rights.^91^ That is not true. The application of proportionality may end up granting, denying or tailoring relief – all depending on the circumstances of the case.

### Current Patent Landscape

Understanding the economic realities and the specificity of technology markets is essential for fully realizing why proportionality is needed and what factors should be considered when it is applied in a particular case. Most of the disputes that give rise to proportionality concerns take place within the ICT, consumer electronics, internet and computer related markets. Many of the considerations and factors discussed are therefore relevant for those markets, but that does not mean that they are not relevant to other markets.

First, at the EPO, there has been a roughly steady growth in the number of patent applications and patents granted in the previous five years, with the exception of last year.^92^ The overall number of patent rights in force globally in 2019 was around 15 million.^93^ Thanks to the ongoing digital transformation, ICT and computer technologies were among the leaders in terms of numbers of patents granted and new patent applications.^94^ The patent landscape is also changing when it comes to the geographical origins of new patent applications, with China and Chinese companies exerting an ever-growing presence.^95^

Second, the large increase in the number of patents, particularly with respect to ICT and computer technologies, has resulted in the creation of patent thickets in those markets.^96^ The formation of such thickets in the ICT and computer technology markets is facilitated by the fact that these markets are rapidly developing, there are numerous companies involved in the innovation and development of new products, and there is significant parallel innovation by multiple, independent producers. The presence of thickets raises the risk of patent disputes. It also makes clearance much more difficult and more costly. It may, for example, require licensing multiple patents from multiple patentees.^97^ Often, full clearance will not be economically feasible, not only because of the number of patents, but also because of the uncertainty surrounding patent validity and the duration of invalidation proceedings. Finally, by raising the cost of acquiring the necessary patents and increasing the likelihood of litigation, thickets may hamper innovation.^98^

Third, products implementing ICT and computer technologies are often complex. They are implementing technologies that are complicated and protected by numerous patents, sometimes numbering in the thousands. They are made of numerous components, often supplied by independent suppliers, which should be responsible for clearing all IP rights that might be reading on a particular product. It is unreasonable to expect that producers of complex products would be able to search and clear all the relevant IP rights.^99^ Even when potentially relevant patent rights are identified, issues of infringement and validity are often questionable. The primary concern of manufacturers of complex products is that there is a high chance of infringing at least one of many patents that reads on the various product components, but it may not be possible to know which one. When costs are sunk and the product has already been designed, an injunction granted to the patentee whose patent reads on a small component of a complex product might be very costly for the product manufacturer. While the additional benefit to the patentee from that injunction over damages in the form of reasonable royalties might be small, the harm to the product manufacturer might be significant.

Fourth, ICT and computer markets often suffer from patent holdup. Holdup occurs when injunction threats are used as leverage to enable the patentee to obtain higher royalties than could normally be obtained if the parties were able to freely negotiate a license and if the infringer was not faced with sunk costs on its investment in the design, manufacture and marketing of a product.^100^ Negative effects of holdup, and even its very existence, are sometimes disputed by those who claim that innovation in those markets undermines claims of holdup. However, evidence from SEP disputes rather seems to suggest that royalties adjudicated in proceedings between implementers and patentees are usually only a fraction of those initially demanded by patentees.^101^

Fifth, ICT, electronics and computer markets are markets with very intense, dynamic competition. This requires significant R&D spending, which unsurprisingly produces a large amount of patentable innovation. As market participants are working on solving similar technical problems, parallel innovation is not uncommon. Patent law is based on a first-to-file principle, but the question is whether parallel and independent innovation could be considered. Should it matter that infringement is not only non-intentional but also that the infringer has independently created an invention, particularly if the invention was created prior to publishing the patent application that is later – once the patent has been granted – asserted as infringed?^102^ It seems that, since proportionality analysis is always very context specific, parallel innovation could at least be considered as a factor when deciding upon the proportionality of injunctive relief.

Finally, it is the presence of non-practicing entities (NPEs) and particularly PAEs or patent trolls that is most problematic.^103^ PAEs represent a narrower concept than NPEs. PAEs are entities that do not produce anything themselves. They are typically not involved in R&D either. Their business model is based on acquiring large portfolios of patents, monetizing them and collecting royalties. PAEs are less interested in transferring and commercializing technology^104^ and are ready to enforce their portfolios through aggressive litigation. However, PAEs usually wait until after the infringer has invested in the patented technology, so the litigation often starts under the circumstances of sunk costs and lock-in.^105^ PAEs risk very little when they enforce their patents since there is no point in infringers countersuing them. Ultimately, PAE critics say that they distort the incentive function of patent law because only a fraction of what they collect as royalties reaches the original inventor.^106^ NPEs are a broader concept. Usually, NPEs other than PAEs or trolls do not manufacture goods but often do conduct their own R&D activities and are active in technology markets, developing their own patented technologies. Universities, research institutes and other scientific institutions can behave as classic examples of NPEs that conduct their own R&D.^107^ Pure technology development companies are another example of NPEs active in the R&D field.^108^

### Proportionality and Patent Injunctions in EU Directive 2004/48

Directive 2004/48 regulates IP enforcement in a comprehensive manner. It is based on the assumption, explicitly articulated in the recitals, that “without effective means of enforcing intellectual property rights, innovation and creativity are discouraged and investment diminished”.^109^ Its comprehensive nature is reflected in its regulation of a whole spectrum of issues, ranging from ensuring effective means for presenting, obtaining and preserving evidence^110^ to effective pecuniary and non-pecuniary remedies. Directive 2004/48 is based on three enforcement principles – effectiveness, dissuasiveness and proportionality.^111^ Whereas the first two might favor rigid enforcement of IP rights, proportionality must be understood in the broader context of how that term has been used in multiple areas of EU law to favor a certain degree of discretion and flexibility^112^ in order to balance competing rights and concerns based on the particular facts of a case, as discussed above.

Since proportionality is always fact specific, remedies – including injunctions – must be applied “in such a manner as to take due account of the specific characteristics of [the] case, including the specific features of each intellectual property right and, where appropriate, the intentional or unintentional character of the infringement”.^113^ Additionally, valuable light is shed on the meaning of proportionality in Art. 3 of Directive 2004/48, which provides that remedies should be “fair and equitable” and “be applied in such a manner as to avoid the creation of barriers […] and to provide for safeguards against their abuse”. Finally, Directive 2004/48 provides that it respects the fundamental rights recognized by the Charter of Fundamental Rights of the European Union.^114^

The concept of the abusive exercise of rights, to which Art. 3(1) of Directive 2004/48 refers, has a long tradition in the national laws of the Member States. As mentioned above, there is substantial consensus as to what constitutes abuse of rights.^115^ Additionally, the concept of abuse of rights was also recognized by the CJEU as one of the general principles of EU law.^116^ Abusive enforcement is thus generally associated with situations that lead to excessively high benefits accruing for the patentee or that cause disproportionate harm to the infringer. Enforcement is also considered abusive when it upsets the legitimate expectations of the infringer and when enforcement is contrary to the economic and social purpose of the enforced rights.

All this leads to the conclusion that the principle of proportionality in Directive 2004/48 is strongly linked with such concepts as fairness and equity.^117^ Additionally, proportionality also points to the need to align enforcement measures, including injunctive relief, with the purpose of patents, namely the stimulation of innovation. The ability to achieve the goals of the patent system often requires flexibility rather than blind rigidity in the application of various enforcement measures. The balancing aspect of the principle of proportionality, especially that of balancing fundamental rights, does not seem to be at the forefront when Art. 3 of Directive 2004/48 is applied. However, it must not be excluded. After all, the Enforcement Directive expressly requires respect for fundamental rights protected by the Charter of Fundamental Rights, and the principle of proportionality remains one of the general principles of EU law recognized by the CJEU.^118^ Certainly, balancing the various fundamental rights – especially the protection of intellectual property and the freedom to conduct business – can assist the courts in fine-tuning their decisions by tilting the balance in favor of denial or tailoring injunctive relief; however, it cannot be the primary tool for making decisions on remedies.

As mentioned above, when assessing the proportionality of patent law remedies, one must consider that, in EU law generally, all IP rights, including patents, are considered as rights granted for a particular purpose.^119^ In the case of patent protection, the goal is to stimulate innovation by providing patentees with an exclusive right to decide on the exploitation of an invention. Remedies in IP law, such as damages or injunctions, are supposed to ensure that IP rights can properly perform their purpose. Whereas damages ensure that the patentee may recoup the investment incurred in R&D activities, injunctions prevent infringers from making use of the patented invention. The availability of injunctive relief allows the patentee to ensure that it retains a technological edge on its competitors. Injunctive relief might also be crucial for adopting a licensing strategy, especially if the patentee envisages granting exclusive licenses. Generally, injunctive relief is a proportionate means for achieving the purpose of patent protection. However, there are situations when the patentee is interested in licensing as many potential licensees as possible and not in enforcing the exclusivity of use of a particular invention. In such cases the patentee is more interested in obtaining monetary compensation for use of the invention. The purpose of patent protection is unlikely to be negatively affected when the patentee receives merely monetary compensation for use of the patented invention.

### Criticism of Proportionality

Recourse to proportionality raises concerns and sometimes vehement criticism. Ohly, whose research has significantly contributed to the understanding of the role of proportionality, admits that injecting flexibility into injunctive relief might pose a threat to the patent system. He believes, however, that the potential threat is not as serious as it might look. This is because the courts – Ohly speaks for the courts in Germany, but that should hold true for courts in other civil law Member States – have applied the abuse of rights doctrine for some time now.^120^

Critics also say that injunctive relief and exclusivity are two sides of the same coin. Without strong injunctive relief – so they say – there will be no exclusive intellectual property rights.^121^ They point to developments that followed the US Supreme Court’s ruling in *eBay*, where Justice Kennedy famously identified patent trolls, described their *modus operandi* and explained the application of the four-factor test for injunctive relief.^122^ Studies have shown that indeed fewer injunctions are granted in cases involving PAEs. Ch. Seaman, who studied how courts reacted to *eBay,* established that PAEs – after prevailing on liability – obtained an injunction in 16% of cases.^123^ Seaman notes that similar observations were made by C. Chien and M. Lemley.^124^

However, to claim that *eBay* undermines the basic character of patents, transforming them from property-like entitlements into liability rules,^125^ seems to be an overstatement. Injunctive relief always remains an option when the infringer is not willing to compensate the patentee for practicing the invention. Thus the negotiations between the patentee and the infringer always take place in the shadow of potential injunctive relief. In the vast majority of cases in markets other than ICT or computer technology, the need for flexibility rarely comes into play, and injunctions are generally available “automatically” once a patent is found to be valid and infringed.

Critics of the *eBay* approach towards injunctive relief – we might assume with reasonable certainty that they would be equally critical of EU-style proportionality – say that decisions like *eBay* encourage the effective infringement of IP rights, including patents. Large market leaders – so the proponents of efficient infringement claim – are encouraged by legal developments such as *eBay*/proportionality to exploit uncertainty surrounding these concepts, and they infringe intentionally rather than conclude a license agreement.^126^ They point to the significant individual and systemic costs resulting from efficient infringement, which might not only undermine individual companies, but also destroy the innovation incentives of the patent system more generally. But this reasoning is highly problematic because it assumes a distorted perception of proportionality. Proportionality analysis always takes into account the character of infringement (whether it is intentional or non-intentional) as well as the nature of technology and mode of exploitation. If the infringement is intentional or even grossly negligent, proportionality analysis would still favor granting injunctive relief against an infringer.

Critics of principles like proportionality say that it gives too much leeway to judges to promote their own policy choices. Some argue it results in uncertainty for the parties as to the outcome, while yet others claim that it is devoid of meaning and should at least be replaced with the standard of gross disproportionality to make its application more predictable. There is some merit to the criticism of uncertainty, but it does not justify discarding the proportionality principle altogether. It does, however, point to the need to identify key factors that would allow courts to conduct the proportionality analysis in a more structured and orderly manner, ensuring its greater predictability, certainty and legitimacy.

### Factors Relevant for the Application of Proportionality to Injunctions in Patent Law

Legal scholars have suggested various factors that could be considered when deciding whether injunctive relief is a proportional remedy.^127^ Ohly for instance proposed that, when applying proportionality to injunctive relief, courts should consider: (1) whether the loss suffered by the infringer from immediately terminating the use of a patented invention would grossly outweigh the benefits to the right holder accruing from an injunction; (2) whether the patentee practices its invention, and whether it is involved in R&D activities or whether its business model concentrates exclusively on collecting royalties; (3) whether the accused technology was developed independently or whether it was copied; (4) whether the infringed patent is strong or weak; and, finally, (5) the degree of fault. These factors certainly cover essential concerns related to the granting of injunctive relief. The application of the fourth factor is problematic. Indeed, the value of patents litigated by PAEs is often questioned. However, the decision whether to grant an injunction is made only after the adjudicating court finds an infringement and – in countries that allow challenges to validity during infringement proceedings – after the court has established validity. Patent weakness is not something that is established during the proceedings. The fourth factor certainly points to a serious problem related to the quality of patents, especially in the ICT, electronics, and computer technology markets,^128^ but it is doubtful whether and how the degree of patent strength/weakness could play a role in an individual case.

N. Siebrasse et al. consider proportionality to be a principle limiting injunctive relief and capable of balancing implementer holdout and patentee holdup concerns.^129^ Generally, the authors recommend that an injunction should not be granted when the negative effects of an injunction are disproportionate to the nature of the infringement and the non-compensable harm that the patentee would suffer if the injunctive relief was not to be granted.^130^ The authors suggest considering: (1) the scale of the infringement in proportion to the whole product, and (2) the relative blameworthiness of the conduct of both infringer and patentee.^131^ Siebrasse et al. also suggest that the relevant conduct of the parties should be considered when deciding about injunctive relief. The infringer’s knowledge of the patents or the fact that the infringer should have known about the patents, or the infringer’s unwillingness to negotiate a license, would favor granting injunctive relief, whereas a patentee’s lack of good-faith engagement in licensing negotiations or acquiescence to infringement and lack of prompt assertion of its rights would all favor denying an injunction.^132^ The authors point, in addition to the negative effects on the enjoined parties, to potential significant effects of an injunction on the manufacturer of a complex product.^133^ Finally, harm to the public might also justify denying injunctive relief, but only when the negative costs exceed those inherent in the functioning of the patent system.^134^

Directive 2004/48 provides rather general guidelines as to how the courts should proceed when assessing the proportionality of injunctive relief. Interestingly, Directive 2016/943 on the protection of trade secrets^135^ provides an expressly formulated list of factors that the courts should take into account before granting an injunction.^136^ Even though protection of trade secrets differs from IP protection, the factors listed in Art. 13 of Directive 2016/943 can certainly be considered when deciding upon the availability and scope of injunctive relief with respect to patents. Article 13 of Directive 2016/943 sheds additional light on the meaning of proportionality in Directive 2004/48.

Formulating a list of factors that could help courts in their application of the principle of proportionality to injunctive relief in patent law would certainly improve the certainty and predictability of outcomes in patent disputes. When drawing up a list of possible factors, it is important to have the following issues in mind. First, the application of proportionality is always context specific and therefore requires careful analysis of the precise circumstances of each case. Second, when deciphering the meaning of proportionality in Art. 3(2) of Directive 2004/48, direct references to the provision of safeguards against abusive enforcement as well as to fairness and equity are indeed crucial. Third, one should also consider that, while patents are granted for a purpose, namely to stimulate innovation, the remedies aimed at protecting these rights should also serve the same purpose. While effective enforcement mechanisms are crucial, they could stifle rather than stimulate innovation if they are excessive. Finally, the application of injunctive relief with respect to patents might affect the following fundamental rights and freedoms: the right to the protection of intellectual property, the right to effective judicial remedy, and the freedom to conduct business. Thus the application of proportionality may require balancing these fundamental rights and freedoms.

Bearing the above considerations in mind, one could identify the following, non-exhaustive, list of factors relevant for assessing proportionality in the patent context: (a) the impact that granting or denying relief could have on the infringer and the patentee; (b) the legitimate interests of the parties; (c) the conduct of the parties; (d) the legitimate interests of third parties; (e) the public interest; and (f) the need to balance various, sometimes competing, fundamental rights and freedoms.

### Application of Factors for Assessing Proportionality of Patent Injunctions

#### Impact of Granting/Denying Relief for Parties

Injunctive relief might have a particularly significant impact on the manufacturers of complex products. This will be the case in particular when a patent reads on a small component – hardly a driver for demand for the complex product.^137^ In such cases, even though the injunction is targeted at cessation of use of a relatively small component and one that does not drive the demand for the product, the manufacturer of a complex product must remove the whole product from the market in order to design around that particular component. Thus, though the value of the complex product to consumers might result primarily from the use of numerous other non-infringing components, consumers might be deprived of a new and innovative product because of a relatively minor infringing use. The harm to the manufacturer of a complex product might be very significant and include sunk costs of the initial product design, costs for removing goods from distribution channels, lost profits on product sales, costs of redesign, and reputational harm. Furthermore, the benefit accruing to the patentee as a result of an injunction and on top of damages may be insignificant. If the patentee does not compete with the manufacturer of a complex product on the market for such products, the injunction would be incapable of ensuring any competitive advantage. The availability of injunctive relief in such cases provides the patentee with significant leverage in negotiations with the infringer, who will be eager to pay higher royalties to avoid sunk costs and other potential harm if an injunction were enforced.

#### Legitimate Interests of Parties

Patentees’ interests in obtaining injunctive relief are very likely to prevail if the patentee commercializes the product by licensing it exclusively to one of its licensees or a selected group of licensees on an exclusive basis. In such cases, depriving a patentee of injunctive relief could impede the licensor’s licensing strategy and undermine its position vis-a-vis current or potential licensees. A patentee will also have a strong interest in obtaining an injunction when it exploits the patented invention itself and that patented solution provides the patentee with a competitive advantage over the remaining market participants.

#### Blameworthiness of Infringer/Patentee

Proportionality should not be used to justify infringements when the infringer acted intentionally or negligently failed to clear the relevant rights. The application of proportionality should neither encourage nor reward intentional or negligent breach. Thus, generally, intentional or negligent infringement should favor the granting of injunctive relief.^138^

However, this raises an important question as to how and when negligence can be established. It would not be justified to expect implementers to undertake unreasonable searches to clear the relevant rights. Such patent searches would be costly and time consuming, and would not often provide much certainty that all relevant rights had been identified or that the identified rights were indeed valid and had been infringed. In the case of complex products in particular, which are often covered by patent thickets, it would not be reasonable to expect the manufacturers of such products to examine each of the components of its product.

Denying injunctive relief is justified if the patentee knowingly waits to assert its patent until the other party incurs significant sunk costs and is locked in to a particular technology.^139^ In such cases the patentee’s behavior is aimed at creating leverage for potential negotiations with the other party in order to obtain excessive royalties. Generally, the fact that the patentee knew about the infringement and knowingly tolerated it without acting promptly to protect its rights works to its disadvantage and justifies denying an injunction.

#### Third-Party and Public Interests

Injunctions generally affect the parties to the proceedings. However, in some cases an injunction might also affect third parties and sometimes even the public at large. This would be the case, for example, if an injunction had an impact on the security of highly popular software^140^ or on access to lifesaving medical devices^141^ or led to the immediate closure of a factory, resulting in the unemployment of a large group of workers.^142^

Public interest concerns should be treated with caution and must weigh against granting injunctive relief in exceptional cases only, that is when the effects significantly exceed the normal costs associated with having a patent system in place. This could be the case, however, when the public would lose access to an entire complex and important product because of the infringement of a trivial feature. One must also remember that there is always a competing public interest in having a patent system capable of stimulating innovation^143^ and that availability of injunctive relief is an important element of that system. In such cases consideration should also be given to tailoring the injunctive relief.

#### Fundamental Rights

Fundamental rights should be balanced after assessing all the factors listed above. Balancing should allow for fine-tuning of the decision on injunctive relief rather than be a substitute for context-specific analysis of the relevant factors. CJEU case law shows that the concept of the fundamental freedom to conduct business allows courts to consider whether burdens imposed on the business – including burdens resulting from remedy enforcement – are excessive. Certainly, the case of complex products is an example where an injunction could be so burdensome that it would amount to a restriction of the freedom to conduct business – one that is unjustified by the need to protect intellectual property as required by Art. 17(2) of the Charter.

#### Tailoring Injunctive Relief

Whereas proportionality may favor monetary relief over an injunction, especially when in the circumstances of the case an injunction would disproportionately harm the infringer, proportionality should also ensure that injunctive relief is not too intrusive upon the interests of the patentee.

The application of proportionality does not leave the court with just two options, namely, to grant or deny injunctive relief. The courts adjudicating in patent infringement proceedings should also consider *tailoring injunctive relief*.^144^ An injunction can be tailored in various ways, depending on the particular circumstances of the case.^145^ For instance, an injunction may be subject to a stay, or its entry into force may be delayed. The purpose behind staying an injunction or delaying its entry into force is usually to allow the user to design around the patented invention while enabling uninterrupted use of a product or service by third parties.^146^ Courts may also tailor the scope of an injunction and exclude certain goods from its scope or allow a sell-off period for certain infringing products. Such tailoring may be required when there is a public interest or an interest of the other party or third parties in ensuring uninterrupted access to certain goods.^147^

The overall effect of tailoring might be especially positive when it induces users to invent around a patented invention. In such cases, tailoring stimulates competition by substitution. This is aligned with the idea that exclusive rights generally stimulate innovation. The purpose of patent exclusivity is not only to provide the right holder with the possibility of recouping its investment in innovation. Patent exclusivity also requires competitors to create new and better solutions to similar technical problems. Thus it requires the substitution of older products by new ones rather than mere imitation. In the end, tailoring while protecting the other party, third parties or public interests may also lead to more innovation and greater dynamic competition.

Tailoring should be the preferred option. While it enables account to be taken of the legitimate interests of the other party, third parties or the public, it also takes into account the freedom of the patentees to decide how their rights are exercised.^148^ Therefore, it is only when the public interest or interests of users cannot be sufficiently safeguarded through the tailoring of injunctive relief that an injunction should be denied.

## Conclusions

It is a great paradox that English courts have fully given up their own concept of *grossly disproportionate* and embraced the proportionality of Directive 2004/48, just to then leave the EU, whereas Germany – the country where the concept originated – has for such a long time remained so sceptical about introducing it into its patent legislation.

However, there is a legal obligation to apply proportionality. Whether we introduce proportionality into patent laws or not, the principle of the indirect effect of EU directives cannot be escaped by Member States’ courts. They are under a clear obligation to interpret their laws in conformity with the EU directives. With proportionality, it is particularly vital because the concept allows for flexibility and greater adaptability to a rapidly changing world. To maintain its global competitiveness and innovation-friendly ecosystem the EU must stay committed to proportionality in IP law in general and with respect to patent remedies in particular.

## Footnotes

1 Kur and Grosse Ruse-Khan (2008), pp. 8–9.

2 Annex 1C Trade-Related Aspects of Intellectual Property Rights to the Marrakesh Agreement Establishing the World Trade Organization of 15 April 1994 (hereinafter TRIPS).

3 Directive 2004/48/EC of the European Parliament and of the Council of 29 April 2004 on the enforcement of intellectual property rights, Official Journal L 157, 30.4.2004 (hereinafter Directive 2004/48, Enforcement Directive, IPRED).

4 For a discussion of the origins of and justification for adopting Directive 2004/48 *see* Strowel and Léonard (2020), pp. 5–7. The authors rightly underline that Directive 2004/48 concentrated on combatting wilful and intentional infringement of intellectual property rights, such as piracy and counterfeiting. With that perspective in mind, it was not surprising that considerations of proportionality were not at the forefront.

5 Malbon, Lawson and Davison (2014), p. 615.

6 Malbon, Lawson and Davison (2014), p. 615.

7 *See*, for example, Art. 11 of Directive 2004/48, which provides that Member States must ensure that right holders are in a position to apply for an injunction against intermediaries whose services are used by a third party to infringe an intellectual property right.

8 C-275/06 *Promusicae*, para. 65.

9 C-484/14 *McFadden*, para. 82.

10 C-70/10 *Scarlet Extended*, para. 48.

11 Miller (2018), p. 238.

12 *See* Love, Helmers, Gaessler and Ernicke (2015), p. 7. The study concerns PAEs’ enforcement activities in Germany and the UK. The data collected shows that PAEs are mostly active in the computer and telecommunications technology sector.

13 Siebrasse et al. (2019), p. 120.

14 Miller (2018), p. 238.

15 Lemley, Shapiro (2007), p. 1993.

16 Merges (2011), p. 161.

17 Ohly (2009), p. 6.

18 Contreras and Husovec (2021), pp. 8–10. The authors show that in most civil law countries – such as Germany, France, Italy, the Netherlands, Finland or Poland – courts have either no or only very little discretion when granting injunctions in patent cases. Even when they have some discretion, it is rarely used, if at all. The authors show that one of the reasons for such a rigid and automatic approach to injunctive relief is the belief that patents are property-like rights that require strong protection of the exclusivity they guarantee.

19 Thomas (2007), p. 39.

20 Merges (2011), p. 159 *et seq*.

21 In C-580/13 *Coty Germany*, the right to information provided for by IPRED (Art. 8) clashed with the banking institutions’ obligation to respect banking secrecy. Fundamental rights such as those to property, to effective judicial remedy and to privacy (secrecy) had to be balanced. The protection of banking secrecy could not be absolute. In *Piotrowski* C-367/16, the CJEU found that the remedies in Polish copyright law did not infringe provisions of EU directives, including Art. 3 of Directive 2004/48, which requires measures to be inter alia proportional. However, the AG found that the proportionality requirement in Art. 3 required that the interests of the user be taken into account. Therefore, it found that a remedy in the form of the right to demand twice the amount of the regular licence fee would be a disproportionate burden on the infringer.

22 Sauter (2013), p. 442.

23 Sauter (2013), p. 446.

24 Scaccia (2019), p. 3.

25 Scaccia (2019), p. 3.

26 Scaccia (2019), p. 3.

27 Scaccia (2019), p. 4.

28 C-11/70 *Internationale Handelsgesellschaft*.

29 Sauter (2013), p. 447.

30 *See*, inter alia, C-19/84 *Pharmon*, C-187/80 *Merck*, C-55 and C-57/80 *GEMA*.

31 C-516/17 *Spiegel Online*, para. 43.

32 C-516/17 *Spiegel Online*, para. 42.

33 Sauter (2013), p. 445.

34 Merges (2011), p. 159.

35 Merges (2011), p. 139.

36 Merges (2011), p. 151.

37 Merges (2011), p. 161.

38 Merges (2011), p. 161.

39 Merges (2011), p. 161.

40 Merges (2011), p. 161.

41 Peukert (2011), p. 67.

42 Dinwoodie and Dreyfuss (2012), pp. 9–10.

43 Dinwoodie and Dreyfuss (2012), p. 9.

44 Contreras (2021), pp. 137–141. The author notices that the lack of patent protection for genomic sequences facilitated research on vaccines and other therapeutic and diagnostic applications based on the viral RNA sequence. If there were patent protection, the author argues, COVID-19 research would be more costly and less efficient.

45 Dinwoodie and Dreyfuss (2012), p. 9.

46 Article 44(1) TRIPS.

47 Malbon, Lawson and Davison (2014), p. 628.

48 Osei-Tutu (2017), p. 224.

49 Article 20.32(1) CETA.

50 Article 20.32(2) CETA.

51 For France *see* Crabb (1964), pp. 4–24; Léonard (2016), pp. 9–29. In France, for example, the doctrine developed as a response to absolutist conceptions of property dominant in French civil law at that time. In the mid-19th century, absolutist conceptions of property were challenged in several cases relating to the exercise of rights to real property. Initially the abuse of rights doctrine was construed in subjective terms, and the finding of abuse hinged on malicious intent to harm a third party by the operations of the owner. This subjective formulation of the abuse of rights doctrine resulted from the doctrine’s roots in tortious liability. The abuse of rights concept was strongly supported by socially oriented jurists, who rejected the ideas of absolutist and individualistic rights. Consequently, they believed that the exercise of rights should be limited by these rights’ social functions rather than by highly individualistic goals. During the second half of the 19th century in some jurisdictions and in the 20th century in others, the abuse doctrine began to provide grounds for challenging the exercise of rights against the rationale or aims (social functions) of those rights. For Germany *see* Gutteridge (1964), pp. 36–39. In the German Civil Code (BGB) the abuse doctrine has its origins in the principle of good faith. In comparison to the French law, however, the BGB codified the abuse of rights doctrine and contains three provisions that are relevant for its application. Article 226 BGB, also referred to as the “*Schikanenverbot*”, states clearly that the right should not be exercised with the sole purpose of causing damage to a third party. It is rather narrowly construed and requires malicious conduct on the part of the right holder. The remaining two provisions cited in the context of abuse of rights are: Art. 242 BGB, a general provision that requires good faith in the execution of obligations; and Art. 826 BGB, which provides for the restitution of damages resulting from activities that are *contra bonos mores*. Courts in Germany have, based on the general provisions of the BGB, found the following abusive: *firstly*, exercise of rights that is grossly inequitable, having regard to the circumstances of the case; *secondly*, exercise of rights when the rights were acquired in violation of the law or in bad faith; *thirdly*, exercise of rights that contradicts prior conduct of the right holder (giving rise to the defense of *venire contra factum proprium*); and *fourthly*, exercise of rights carried out in violation of Art. 226 BGB, namely with the intent to harm third parties.

52 Siebrasse et al. (2019), p. 142.

53 Siebrasse et al. (2019), p. 142.

54 Ohly (2021), p. 305. The author also shows that Sec. 242 German Civil Code (BGB), which refers to the principle of good faith, is already a statutory basis for the courts in trademark and unfair competition cases for granting a period of grace. Such a grace period is one example of how injunctive relief can be tailored.

55 Directive 2001/29/EC of the European Parliament and of the Council of 22 May 2001 on the harmonisation of certain aspects of copyright and related rights in the information society, Official Journal L 167, 22.06.2001 (hereinafter Directive 29/2001).

56 Directive 2000/31/EC of the European Parliament and of the Council of 8 June 2000 on certain legal aspects of information society services, in particular electronic commerce, in the Internal Market (‘Directive on electronic commerce’), Official Journal L 178, 17.07.2000 (hereinafter E-commerce Directive, Directive 31/2000).

57 *Promusicae*, para. 29.

58 *Promusicae*, paras. 30–31.

59 *Promusicae*, paras. 61–63.

60 *Promusicae*, para. 68.

61 *Promusicae*, para. 66; Directive 2002/58/EC of the European Parliament and of the Council of 12 July 2002 concerning the processing of personal data and the protection of privacy in the electronic communications sector (Directive on privacy and electronic communications), Official Journal L 201, 31.07.2002.

62 *Promusicae*, para. 70.

63 *Scarlet Extended*, paras. 15–16.

64 *Scarlet Extended*, para. 17.

65 *Scarlet Extended*, para. 23.

66 *Scarlet Extended*, para. 25.

67 *Scarlet Extended*, para. 26.

68 *Scarlet Extended*, para. 28.

69 *Scarlet Extended*, para. 40.

70 *Scarlet Extended*, para. 36.

71 *Scarlet Extended*, para. 43.

72 *Scarlet Extended*, para. 48.

73 *Scarlet Extended*, paras. 50–51.

74 *UPC Telekabel*, paras. 11–12.

75 *UPC Telekabel*, para. 14.

76 *UPC Telekabel*, para. 15.

77 *UPC Telekabel*, para. 47.

78 *UPC Telekabel*, para. 49.

79 *UPC Telekabel*, para. 50.

80 *UPC Telekabel*, para. 51.

81 *UPC Telekabel*, para. 53.

82 *UPC Telekabel*, para. 56.

83 *McFadden*, paras. 22–23.

84 *McFadden*, para. 82.

85 *McFadden*, para. 85.

86 *McFadden*, para. 87.

87 *McFadden*, para. 88.

88 *McFadden*, para. 91–92.

89 *McFadden*, para. 93.

90 Geiger and Izyumenko (2019), p. 15.

91 Merges (2017), pp. 22–23. The author argues that *eBay* did not change the nature of patent rights and that injunctions are still granted almost automatically in the majority (over 75%) of cases. Merges sees *eBay* as weakening patents only in those cases where an injunction is capable of causing serious economic damage.

92 EPO Patent Index 2020 (2021), pp. 2, 8.

93 WIPO IP Facts and Figures 2020 (2021), p. 9.

94 EPO Patent Index 2020 (2021), p. 4; WIPO IP Facts and Figures 2020 (2021), p. 15.

95 WIPO IP Facts and Figures 2020 (2021), p. 12.

96 Shapiro (2001), p. 120.

97 Shapiro (2001), p. 120; Melamed and Lee (2016), p. 17. The authors claim that pre-clearance is still possible in sectors such as pharmaceuticals and biotechnology, where the number of patents still allows patent searches to be conducted.

98 Shapiro (2001), p. 121.

99 Merges (2010), p. 1591.

100 Lemley and Shapiro (2007), p. 1993.

101 Letter of 77 former governmental officials and professors of law, economics and business to Assistant Attorney General Makham Delrahim, 17 May 2018.

102 Liivak (2009), p. 10. The author suggests that a patent system based on copying could address some of the troll related problems. For the purposes of this analysis the question of course is only whether independent creation could be a factor for consideration when applying proportionality.

103 Scholars and government agencies have been trying to grasp the phenomena of PAEs and NPEs publishing numerous studies about their activities. They provide different typologies of PAEs and NPEs. Catropia, Kesan and Schwartz, for example, identify the following types of patent holders that assert their patents: universities, individual inventors, failed start-ups, technology development companies, patent holding companies, mass patent aggregators, and operating companies. They add that costs and benefits for the particular group of patent asserters are very different. *See* Catropia, Kesan and Schwartz (2014), pp. 659–660. Miller identifies 13 types of entity that assert their patents. *See* Miller (2018), p. 244. Allison, Lemley and Schwartz propose five distinct categories of entity that assert their patents: operating company, university, individual, failed start-up/failed operating company and patent holding company (including aggregators). *See* Allison, Lemley and Schwartz (2017), p. 249.

104 Miller (2018), p. 238.

105 Merges (2010), p. 1591.

106 Miller (2018), p. 239.

107 Lemley (2008), p. 611 *et seq*. Lemley makes an interesting point that an entity is a troll if it acts like troll. The issue therefore is not whether universities are trolls but whether they act as trolls.

108 Cotropia, Kesan and Schwartz (2014), p. 658.

109 Recital 3(2) of Directive 2004/48.

110 Articles 6–8 of Directive 2004/48.

111 Article 3 of Directive 2004/48.

112 Sikorski (2019a, b), p. 33.

113 Recital 17 of Directive 2004/48.

114 Recital 32 of Directive 2004/48.

115 Siebrasse et al. (2019), p. 142.

116 C-321/05 *Kofoed*. Case cited after Losurdo (2009), pp. 10–12.

117 Husovec (2020), p. 5.

118 Recital 32 of Directive 2004/48.

119 Ullrich (2013), pp. 10–11. Peukert (2011), p. 67. Peukert shows that though the declared purpose of IP rights is to stimulate innovation and creativity, IP rights have become an end in themselves.

120 Ohly (2020), p. 306.

121 Epstein (2010), p. 494, cited after Merges (2017), p. 22.

122 *eBay Inc. v. MercExchange*, 547 U.S. 388 (2006). Justice Kennedy famously said: “An industry has developed in which firms use patents *not as a basis for producing and selling goods* but, instead, *primarily for obtaining licensing fees*”. He went on to explain that “[f]or these firms, an injunction, and the potentially serious sanctions arising from its violation, can be employed as *a bargaining tool to charge exorbitant fees* to companies that seek to buy licenses to practice the patent”. He then explained how injunctions might be used in the context of complex products: “When the patented invention is but *a small component of the product* the companies seek to produce and the threat of an injunction is employed simply for *undue leverage* in negotiations, legal damages may well be sufficient to compensate for the infringement and an injunction may not serve the public interest. In addition, injunctive relief may have different consequences for the burgeoning number of patents over business methods, which were not of much economic and legal significance in earlier times”. Finally, he also showed why flexibility was needed on rapidly developing markets: “The *equitable discretion* over injunctions, granted by the Patent Act, is well suited to allow courts to *adapt to the rapid technological and legal developments* in the patent system” [emphasis added].

123 Seaman (2014), p. 1988.

124 Chien and Lemley (2012), p. 10.

125 Seaman (2014), p. 1983.

126 Mossoff and Viswanathan (2017). Explaining Efficient Infringement.

127 Ohly (2009), pp. 8–9.

128 On the notion of weak patents, probabilistic patents, and the uncertainties surrounding them, *see* Farrell and Shapiro (2008), pp. 1347–1348; Lemley and Shapiro (2005), pp. 9–14.

129 Siebrasse et al. (2019), p. 145.

130 Siebrasse et al. (2019), p. 146.

131 Siebrasse et al. (2019), p. 147.

132 Siebrasse et al. (2019), p. 147.

133 Siebrasse et al. (2019), p. 149.

134 Siebrasse et al. (2019), p. 150.

135 Directive (EU) 2016/943 of the European Parliament and of the Council of 8 June 2016 on the protection of undisclosed know-how and business information (trade secrets) against their unlawful acquisition, use and disclosure, OJ L 157, 15.6.2016, 1–18 (hereinafter Directive 2016/943).

136 Article 13 of Directive 2016/943. These are: (a) the value or other specific features of the trade secret; (b) measures taken to protect the trade secret; (c) the conduct of the infringer in acquiring, using or disclosing the trade secret; (d) the impact of the unlawful use or disclosure of the trade secret; (e) the legitimate interests of the parties and the impact which the granting or rejection of the measures could have on the parties; (f) the legitimate interests of third parties; (g) the public interest; and (h) the safeguarding of fundamental rights.

137 Ricolfi (2015), p. 165.

138 Siebrasse et al. (2019), p. 148.

139 Ohly (2021), p. 308.

140 *Z4 Technologies, Inc. v. Microsoft Corp*., 434 F. Supp. 2d 437 (E.D. Tex. 2006).

141 *Edwards Lifesciences LLC v. Boston Scientific SCIMED INC*, [2018] EWHC 1256 (Pat).

142 *Bayer Intellectual Property GmbH v. Ajanta Pharma Ltd.*, IA No.86/2017, Hon’ble Mr. Justice R.k.Gauba, Order 4.01.2017.

143 *Polara Engineering, Inc. v. Campbell Co.*, 237 F. Supp. 3d 956 – Dist. Court, CD California 2017.

144 Chien and Lemley (2012), pp. 28–32; Golden (2012); Chien and Schulman (2018), pp. 247–250.

145 For the most recent comprehensive analysis of various possible forms of tailoring of injunctive relief *see* Contreras and Husovec (2021), pp. 16–17. The authors discuss the following forms of tailoring: sell-off periods, delays and carve-outs. These various forms of tailoring guarantee uninterrupted access to essential products such as medicines or medical devices as well as to critical infrastructure.

146 *See*, for instance*, Edwards Lifesciences LLC v. Boston Scientific SCIMED INC*, [2018] EWHC 1256 (Pat). In that case, (then) Justice Arnold decided to stay an injunction for a period of 12 months with the possibility for extension on the ground that an injunction without stay would deprive patients of access to the lifesaving transcatheter heart valve (THV) Sapien 3, used in the treatment of patients with aortic stenosis. Staying the injunction was necessary for re-training medical personnel to use another device.

147 Siebrasse et al. (2020), p. 155.

148 Sikorski (2019a, b), pp. 41–42; Ullrich (2013), p. 22.
